# A Cross-Sectional Study of Salivary Cortisol, Alpha Amylase, and Measures of Psychological Distress in Children Undergoing Dental Procedures

**DOI:** 10.3390/children12091235

**Published:** 2025-09-16

**Authors:** Shelby Main, Stephen Suchy, Marcela Carrilho, Zinat Sharmin, Sheila Hall, Jahnavi Rao, Caroline M. Sawicki, Ketlen Bystrom, Linda Sangalli

**Affiliations:** 1 College of Biomedical Science, Midwestern University, Downers Grove, IL 60515, USA; shelby.main@midwestern.edu; 2 College of Dental Medicine, Midwestern University, Downers Grove, IL 60515, USA; ssuchy@midwestern.edu (S.S.); mcarri@midwestern.edu (M.C.); zsharm@midwestern.edu (Z.S.); shall1@midwestern.edu (S.H.); jrao@midwestern.edu (J.R.); kbystr@midwestern.edu (K.B.); 3 Department of Pediatric Dentistry and Dental Public Health, University of North Carolina, Chapel Hill, NC 27599, USA; caroline_sawicki@unc.edu

**Keywords:** biomarkers, salivary alpha amylase, dental stress analysis, dental anxiety, pediatric patients, dental care for children

## Abstract

Background/Objectives: Dental fear and anxiety are areas of concern in clinical pediatric dentistry, often leading to treatment avoidance and negative oral-health consequences. Cortisol and alpha amylase, measurable in saliva, have been proposed as biomarkers of stress and may provide an objective means of assessing and monitoring distress over time. This study examined measures of psychological and physiological distress in pediatric patients undergoing dental procedures and their correlation. Methods: 7-to-17-year-old new patients scheduled for a dental procedure or orthodontic bonding completed a psychological battery assessing dental fear (using Children’s Fear Survey Schedule—Dental Subscale), stress (Perceived Stress Scale), and dental anxiety (Modified Children Dental Anxiety Scale). Before the dental appointment, we assessed anticipated pain intensity, heart rate (HR), and collected two saliva samples to quantify cortisol and alpha amylase. Correlations between psychological and physiological measures were assessed with Pearson’s correlation and treatment groups were compared with independent *t*-tests. Results: Out 34 participants (12.8 ± 2.7 y/o, 52.9% females, 73.5% pediatric patients and 26.5% orthodontic patients), 38.2% endorsed moderate anxiety; 85.3% reported moderate to high stress; and 29.6% indicated dental fear. Psychological distress was not influenced by procedure type. Dental fear positively correlated with dental anxiety (*p* < 0.001) and HR (*p* < 0.001); dental anxiety positively correlated with anticipated pain (*p* = 0.010) and HR (*p* = 0.003); stress positively correlated with HR (*p* = 0.006). Even if 72.2% of participants had cortisol value outside normal range and those with greater stress exhibited elevated cortisol, cortisol and alpha amylase levels (measured on n = 18) were not correlated with psychological variables. Conclusions: Cortisol and alpha amylase levels were not correlated with psychological measures in a cross-sectional study on pediatric patients undergoing dental procedures.

## 1. Introduction

Many acute stressful experiences are not necessarily harmful, yet can be associated with pain, anxiety, and distress [1]. Dental procedures fall into this category of acute stress responses, particularly in children, where baseline psychosocial distress can influence pain and anxiety experienced during the procedure more than the procedure itself [2].

Dental fear and dental anxiety continue to be major areas of concern in clinical pediatric dentistry. Approximately 30% of children between the ages of 2 to 6 years old experience dental fear and anxiety, especially at their first dental visit [3]. This prevalence tends to decrease over time, accounting for about 15% among the adult population [4]. Despite decreasing with age [5,6,7,8], these conditions not only complicate treatment delivery due to patients’ non-compliance and uncooperativeness during dental procedures in [9], but also contribute to avoidance of dental care [1]. For example, studies have found that patients admitting symptoms of dental fear are three times at higher risk of missing their dental appointment compared to patients without dental fear [9]. As a result, this avoidance can have long-term negative effects for both oral health (e.g., increased number of missing teeth, pain associated with untreated caries, reduced access to dental care [10,11]), and overall well-being (e.g., loss of disability-adjusted life years [12], negative impact on quality of life [13]), among others. Therefore, the ability to identify and quantify psychological distress could better equip the pediatric dentists to anticipate and manage the behaviors driven by dental fear.

Self-reported instruments remain the standard for assessing dental fear and anxiety; however, their accuracy in younger children may be influenced by parental presence [14], limited comprehension, and recall bias. For example, some assessment tools for psychological distress referred to prior experience (i.e., in the past week), thus potentially limiting reliability and not necessarily correlating to the present situation. Moreover, they are not routinely utilized in pediatric dentistry clinical practice.

To reduce these limitations derived from purely self-reported psychological assessment, incorporating physiological measures can provide more comprehensive and objective assessments. Quantification of physiological measures allows for monitoring acute and rapidly changing outcomes, providing valuable real-time insights into stress response. Recent advances in salivary diagnostics to quantify salivary-based molecular biomarkers have significantly expanded this field of research [15], as an alternative for diagnosis and monitoring disease progression [16]. Salivary diagnostics offer numerous benefits, such as being less invasive, cost-effective, hygienic, and is particularly suitable for pediatric populations.

While the adult literature has consistently pointed to salivary cortisol as a reliable stress-related biomarker, few studies have explored the relationship between psychological and physiological measures of distress in children, yielding contradictory results. For example, some studies have claimed the concentration of salivary cortisol to be greater in returning patients compared to new patients and to correlate with higher state of anxiety, showing correlation of psychological and physiological stress [17,18,19]. Sex differences were observed amongst children in stress symptoms, with greater severity in females [17,19,20]. Another potential molecule of interest is salivary alpha amylase, which levels were found to be positively correlated with dental fear, especially in children undergoing dental procedures with local anesthetics [21] and dental anxiety [22]. At post-treatment, but not before the procedure, all salivary measures were significantly correlated with dental anxiety [18]. Another study corroborated the difference between new and returning patients [19], and found that returning patients experienced greater level of stress, most likely from previously being exposed to the dental environment [19]. While most research has focused on children undergoing dental treatment, far less is known about stress responses in those initiating orthodontic therapy [17]. Orthodontic bonding is a common procedure that involves prolonged chair time and potential discomfort despite not requiring the use of vibrating instrument nor local anesthesia, but whether it induces comparable levels of anxiety and physiological stress as routine dental procedures is unknown.

This study had the following three aims. The primary aim was to examine the psychological distress among pediatric patients scheduled to undergo dental treatments or orthodontic bonding at a large predoctoral dental school. A secondary aim was to investigate and quantify measures of physiological distress (i.e., salivary cortisol and alpha amylase) in pediatric patients before starting their dental procedure. A tertiary aim was to explore the correlation between psychological distress and acute physiological stress in this patient population. We hypothesized that psychological distress would be correlated with increased measures of acute physiological stress. Additionally, we hypothesized that both measures of psychological and physiological distress would be greater in pediatric patients undergoing dental procedures compared to orthodontic patients starting orthodontic therapy.

Findings from this study will deepen our understanding on the identification of useful stress-related molecules that could be utilized as stress-related biomarkers during dental management of pediatric patients. Ultimately, results from the proposed project are expected to have significant implications for pediatric dentistry by highlighting the importance of managing dental anxiety and fear to improve overall patient outcomes.

## 2. Materials and Methods

The Strengthening the Reporting of Observational Studies in Epidemiology (STROBE) statement checklist was used for reporting this observational research [23].

### 2.1. Study Design

This cross-sectional study was approved by the Institutional Review Board (IRB) of Midwestern University (CIRB#IL24015). The study was registered at ClinicalTrials.gov (NCT06730425).

### 2.2. Study Size

In line with a similar study [24], the sample size was calculated based on the correlation between dental anxiety and cortisol. Assuming a large effect size (r = 0.5), alpha = 0.05, and power = 0.80, a total number of 29 participants would be enough to reveal statistically significant associations.

### 2.3. Setting

Participants were recruited over an 8-month period (from September 2024 to April 2025) from pediatric patients referred to the pediatric or orthodontic clinic at the Multispecialty Clinic of the College of Dental Medicine—Illinois (Midwestern University, MWU).

### 2.4. Participant Recruitment and Eligibility

Eligible participants were healthy pediatric patients (between 7 and 17 years old) presenting as new patients to the pediatric or orthodontic clinic at the MWU Multispecialty Clinic; able to read and speak English language; willing to complete the study tasks (see Section 2.5 below); and scheduled for a second appointment within 2 weeks to undergo a dental procedure or start an orthodontic treatment.

Exclusion criteria were adult patients (≥18 years old); those unable to read and speak English language; returning patients (i.e., those with prior visits to the pediatric or orthodontic departments at MWU); patients with systemic or endocrine disorders known to affect salivary flow (e.g., diabetes mellitus, Sjogren’s syndrome, renal disease, hepatic disease, pancreatic insufficiency, thyroid dysfunction); patients with a history of head and neck radiation or salivary gland surgery; patients currently using medications known to alter salivary flow (e.g., anticholinergics, diuretics, antihypertensives, etc.); patients with a diagnosed psychiatric disorder or mental illness affecting study participations; patients with active oral mucosal disease (e.g., candidiasis, lichen planus) or acute infections that could temporarily affect salivary secretion. 

Due to differences in patient flow between the pediatric and orthodontic clinics and organizational constraints, we implemented an allocation strategy of approximately 3:1 between participants recruited from the pediatric clinic and those recruited from the orthodontic clinic.

### 2.5. Study Procedure

**Figure 1:**
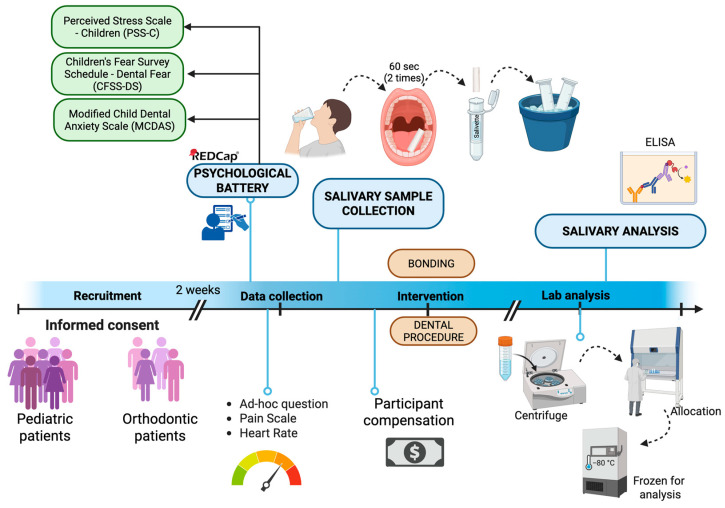
Study procedure.

Figure 1 illustrates the study procedure:

- Baseline visit (T0): After confirming the eligibility criteria from reviewing the patient’s electronic medical record, including medical and surgical history and medication, the study personnel explained both eligible participant and parental guardian the study purpose, type of information collected, benefits, and risks of participating, study tasks, as well as the estimated time for completion and compensation for participating. An informed consent and assent were obtained from parents/guardians and pediatric patients, respectively. Participants were then scheduled to return for a second visit within the following 2 weeks to either undergo the dental procedure or start the orthodontic treatment. Participants were instructed to not eat or drink one hour prior to the second appointment, to not influence salivary sample analysis.
- Dental appointment (T1): All study-related tasks were conducted and completed before starting any dental procedures. Enrolled participants received an electronic device (Ipad, Apple 9th gen, Apple Inc., Cupertine, CA, USA) and were asked to complete a psychological battery of validated questionnaires through the electronic software REDCap^TM^ (Vanderbilt University, Nashville, TN, USA). This baseline psychological battery assessed dental fear (through the Children’s Fear Survey Schedule—Dental Subscale, CFSS-DS), dental anxiety (through the Modified Child Dental Anxiety Scale, MCDAS), and self-perceived stress (through the Perceived Stress Scale for Children, PSS-C, see Section 2.6 below). To best limit potential confounders derived from the presence of the parents, the same study personnel assisted the participants during the administration of the questionnaire. Pilot study suggested that the time to complete this baseline battery was approximately 10–15 min.

Before undergoing the dental procedure (i.e., pediatric patients) or bonding the orthodontic appliance (i.e., orthodontic patients), the study personnel collected self-reported current and anticipated pain intensity level (on a 0–10 Visual Analogue Scale, VAS), self-perceived stress and anxiety levels, and sleep quality with ad hoc questions (see Section 2.6 below). In addition, heart rate was also measured via a pulse oximeter (Beijing Choice Electronic Technology, Beijing, China). After confirming that the patient has refrained from eating for at least 1 h before the procedure, a salivary collection was performed as described below (see Section 2.7).

Participants were compensated with a $20 gift card after completing all the study tasks.

### 2.6. Psychological Measures

Participants were asked to complete the following psychological battery in the dental chair, while the parent/guardian remained in the waiting room to not influence the responses (Table 1).

#### 2.6.1. Self-Perceived Stress

Self-perceived stress was assessed through the Perceived Stress Scale for Children (PSS-C), a 14-item questionnaire presenting different scenarios potentially evoking a mental state of stress [25]. Answer options were based on a 4-point Likert scale, with anchors from 0 = “Never” to 3 = “A lot”. Total score was obtained by summing all items, and ranged between 0 and 39, with higher scores identifying greater stress symptomatology. According to existing literature, values between 0 and 13 were identified as *low stress symptoms;* values between 14 and 26 as *moderate stress symptoms;* and values above 27 as *high stress symptoms* [25]. This questionnaire has been validated in various populations including dentists, healthcare students, and pediatric patients [26,27,28] and is designed to distinguish between general and dental-related stress [28].

#### 2.6.2. Dental Anxiety

Dental anxiety was assessed through the Modified Children Dental Anxiety Scale (MCDAS), an 8-item, 5-point Likert scale (from 1 = “Relaxed/not worried” to 5 = “Very worried”) inquiring about dental situations known to influence anxiety [29]. A total score was obtained by summing all items, and ranged between 8 and 40, with higher scores identifying greater dental anxiety symptomatology. Specifically, values from 8 to 19 identified *absence of dental anxiety*; values between 19 and 30 identified *moderate dental anxiety*; and values > 31 identified *severe dental anxiety*. This questionnaire has been validated in pediatric patients [30].

#### 2.6.3. Dental Fear

Dental fear was measured via Children’s Fear Survey Schedule—Dental Subscale (CFSS-DS), a 5-point Likert scale ranging from 1 = “Not afraid at all” to 5 = “Very much afraid”. The CFSS-DS consists of 15 items, each representing hypothetical scenarios occurring during a dental procedure, such as the sounds of dental instruments or doctors in white coat. A total score was obtained by summing each item and ranged between 15 and 75, with higher values identifying greater dental fear symptomatology. Values < 38 reflected *absence of dental fear*, while values equal or greater than 38 reflected *presence of dental fear*.

#### 2.6.4. Additional Psychological Measures

In addition to the above-mentioned validated questionnaires, the participants were asked to rate their sleep quality the night prior (“How well did you sleep last night?”, with Likert-scale answer options from 1 = “Worst quality” to 5 = “Best quality”), current fear (“How do you rate your level of fear right now?”, with Likert-scale answer options from 1 = “Not afraid” to 5 = “Extremely afraid”), and current stress (“How do you rate your level of stress right now?”, from 1 = “No stress” to 5 = “Extreme stress”). Given that psychological distress has also been associated with self-reported pain outcomes, current (i.e., pain right now) and anticipated (i.e., pain that the participant expected to feel during the procedure) pain intensity levels were assessed using the Visual Analogue Scales, from 0 identifying “No pain” to 10 identifying “Extreme pain”. Adhering to the Wong-Baker Pain Rating Scale [31], the lowest number (0) paired with a happy face and the highest number paired with a crying face. Higher levels of pain intensity have been suggested among children with more severe dental anxiety [32].

### 2.7. Physiological Measures

To reduce the limitations derived from a purely psychological assessment, we incorporated the collection of physiological measures of cortisol and alpha amylase through salivary collection. We also quantified measures of Immunoglobulin A (IgA) serving as a control; however, data was omitted as inconclusive and not reliable. Further details are available in Supplementary File S1.

#### 2.7.1. Saliva Sample Collection

Salivary sample was collected through absorption technique with the use of Salivettes^®^ (Nümbrecht, Germany) collection devices due to its reliability and minimally orally invasiveness for our participant age range. Salivette^®^ device contains a sterile cotton swab inside a labeled conical tube. After confirming that the participant had restrained from food intake in the previous hour, participants were first asked to gargle water for 1–5 s, 10 min prior to saliva collection. Next, participants were instructed to place the swab in their mouth for 60 s, while chewing. The same procedure was performed to collect the second sterile cotton swab. Finally, the study personnel instructed the participants to return the saliva-absorbed cotton swab into the Salivette^®^ tube, which was closed immediately with a dedicated stopper. According to prior piloting of the quantity of saliva volume necessary for adequate salivary analysis of salivary cortisol and alpha amylase, two Salivette^®^ were collected per participant. The Salivette^®^ tubes containing saliva samples were externally wiped with 0.1% sodium hypochlorite and placed immediately in a Styrofoam ice bath. The Salivette^®^ tubes were labelled with the record ID assigned by REDcap^TM^ for that specific participant.

#### 2.7.2. Saliva Sample Storage

Samples remained in Styrofoam ice bath immediately after collection to prevent endogenous or bacterial degradation of the sample. Saliva samples were then processed through centrifuge (1000× *g*, 2 min), and then aliquoted in conical tubes, each containing 100 uL. They were then stored in the research facilities freezer at −80 °C.

#### 2.7.3. Bicinchoninic Acid (BCA) Assay

Total salivary protein concentration to measure protein concentration amongst two Salivettes^®^ was determined using a BCA assay (ThermoFischer Scientific, Waltham, MA, USA) following the manufacturer’s protocol. Protein standards were prepared from serum albumin (BSA, 0–2000 μg/mL). Samples were plated in triplicate, incubated at 37 °C and absorbance was measured at 562 nm on a Promega GloMax Discover (Promega Corporation, Madison, WI, USA) plate reader. Protein concentrations were calculated from standard curves and used to normalize ELISA results.

#### 2.7.4. Salivary Cortisol and Alpha Amylase ELISA

Cortisol concentrations were quantified using commercially available ELISA kits (Invitrogen-ThermoFisher Scientific, Frederick, MD, USA, and Biomatik, Kitchener, ON, Canada) according to the manufacturer’s instructions. All samples were run in triplicate and absorbance was recorded at 450 nm on a Promega GloMax Discover plate reader. Dilution factors were determined in pilot experiments as corresponding to 1:8 for cortisol and 1:50,000 for alpha amylase. Further details on the protocols used in the current study are available in Supplementary File S1.

#### 2.7.5. Normalization Process

The process of data normalization consisted of calculating the concentration of salivary molecules detected by the immunoassay experiments (cortisol, alpha amylase) as a function of the total protein concentration which was quantified through the BCA Assay. The purpose of including data normalization in our study was to properly correlate and make comparisons between participants. Dividing values extracted from saliva allowed us to numerically identify concentration values across saliva samples. Thus, expressing the salivary molecules concentration over total protein values indicates the total cortisol and alpha amylase values relative to each participant’s total salivary protein content. Variability was taken into consideration amongst experiments on each protein between different saliva samples and corrected for any discrepancies. All concentrations were converted to μg/mL and were calculated to determine the exact value at fixed time (prior to dental procedure at T1) of cortisol and alpha amylase within participant’s total salivary protein concentration.

### 2.8. Quantitative Variables and Statistical Analysis

Before conducting the analysis, variables were assessed for missing data and outliers (defined as values exceeding 3 standard deviations from the mean). Missing or unusable samples were excluded listwise.

Normality distribution of continuous data was assessed with Shapiro–Wilk test and non-normally distributed data (i.e., self-perceived stress) underwent logarithmic transformation to favor parametric tests.

The two groups of pediatric and orthodontic participants were compared on psychological and physiological variables with independent *t* tests and chi-square tests, as appropriate. Effect sizes (Cohen’s *d*, Cramer’s V, and phi) were calculated as appropriate and interpreted according to established thresholds.

Given that they did not significantly differ, the two groups were aggregated together to test the aims of the study. The values of the salivary molecules were first compared with established reference values according to sex and age groups. Specifically, salivary cortisol levels of 0.0102–0.0273 μg/mL and alpha amylase levels of 40–400 μg/mL were considered within the normal range according to published literature [33].

Given that dental anxiety can be categorized in three categories of absence (for values from 8 to 19 on an 8–40 scale), moderate (for values from 19 to 30 on an 8–40 scale), and severe dental anxiety (for values above 31 on an 8–40 scale), levels of the two salivary molecules were compared between the three groups with one-way analysis of variance (ANOVA). Similarly, levels of the two salivary molecules were compared according to the levels of self-perceived stress (low stress for values below 13 on a 0–40 scale; moderate stress for values between 14 and 26 on a 0–40 scale; and high stress for values above 27).

Finally, the levels of the two salivary molecules of interest (expressed as continuous variables) were correlated with the levels of psychological distress of dental anxiety, dental fear, and self-perceived stress with Pearson’s correlation. As the psychological measures have established cut-off points, analyses were repeated by considering psychological measures as categorical variables.

Data was analyzed with the software program for statistical analysis SPSS, Statistical Package for the Social Sciences (IBM, SPSS Inc, v29, IBM Corp., Armonk, NY, USA). For all analyses, the *p* value was set at α < 0.05.

## 3. Results

**Figure 2:**
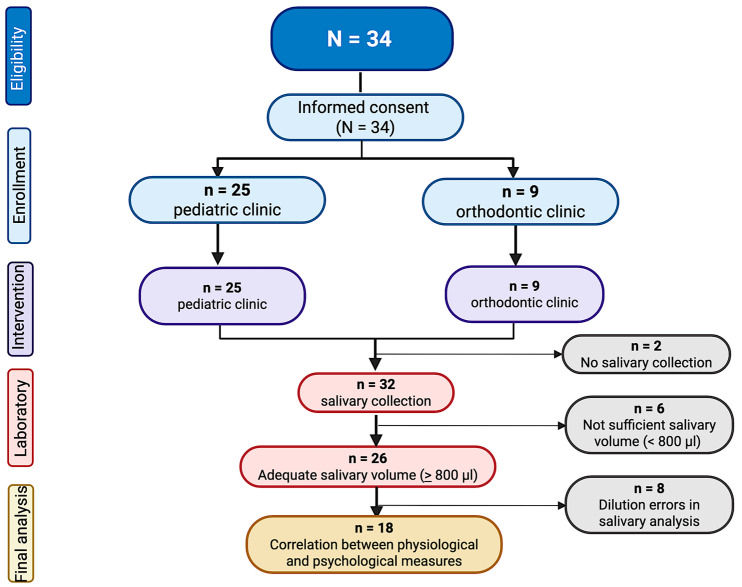
Flowchart of study participants.

A total of 34 participants were recruited for this study (12.8 ± 2.7 years old, 52.9% females), being 25 from the pediatric clinic and 9 from the orthodontic clinic (Figure 2). Majority of participants were Caucasian (79.4%) and non-Hispanic (67.6%). Of the participants scheduled in the pediatric clinic, 40.9% were scheduled for cleaning, 31.8% for composite fillings, 13.6% for a pulpotomy, and 4.5% for a crown. Overall, 32.0% of pediatric dental procedures required the use of anesthetics. As no significant differences in psychological and physiological variables were found across procedure types or anesthetic use, the pediatric sample was analyzed as a single group. Table 2 shows the demographic characteristics of all the participants, as well as the comparison between pediatric and orthodontic patients.

### 3.1. Psychological Variables of Distress

**Figure 3:**
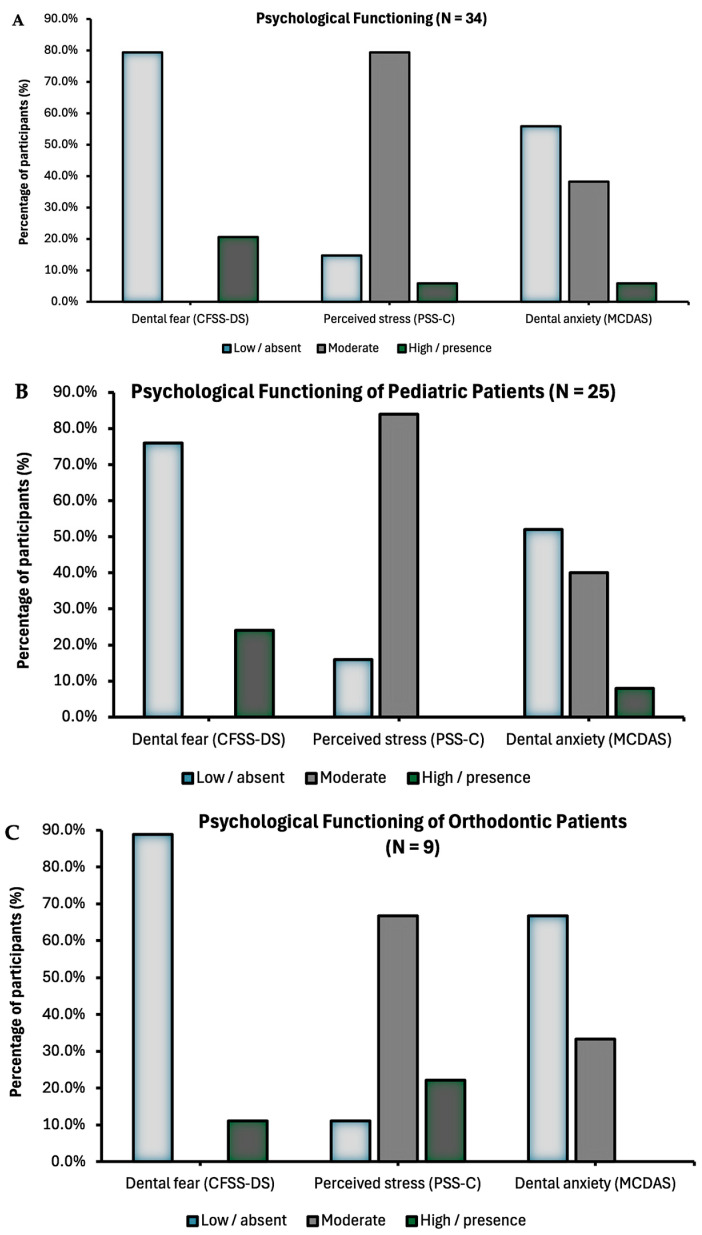
Psychological functioning of the entire sample (**A**), pediatric patients (**B**), and orthodontic patients (**C**). Note: CFSS-DS: Children’s Fear Survey Schedule—Dental Subscale, MCDAS: Modified Children Dental Anxiety Scale, PSS-C: Perceived Stress Scale—Children.

Psychological data were collected in all the 34 participants (Table 2). About 79.4% of the pediatric patients reported moderate stress, while 38.2% and 20.6% reported symptoms of dental anxiety and dental fear, respectively (Figure 3A). Only 5.9% of participants scored at high perceived stress and severe anxiety.

**Figure 4:**
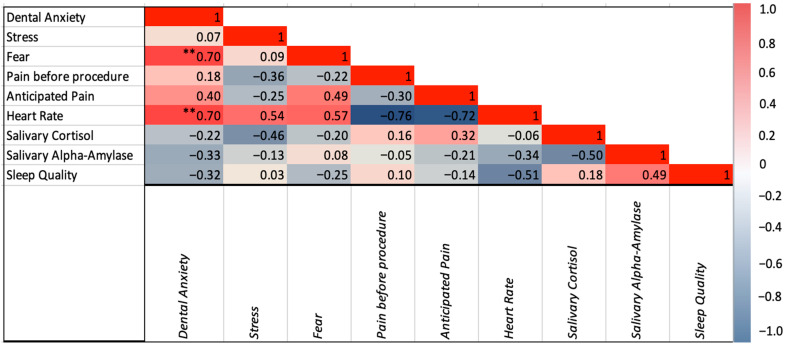
Correlation between psychological and acute physiological variables. ** *p* < 0.001.

Pediatric patients undergoing dental procedures or starting an orthodontic treatment did not significantly differ in perceived stress, dental anxiety, and dental fear (all *p*’s > 0.05, Table 3 and Figure 3B,C). However, pediatric patients reported significantly higher pain before the procedure compared to the orthodontic patients (20.6 ± 1.1 vs. 0.0 ± 0.0, *p* = 0.015). Among the pediatric patients, pain before the procedure did not significantly differ across participants scheduled for different type of procedures. Psychological variables of distress did not significantly differ according to sex (all *p*’s > 0.05). Dental anxiety was positively correlated with fear (r(34) = 0.697, *p* < 0.001), anticipated pain (r(27) = 0.395, *p* = 0.042), and heart rate (r(25) = 0.695, *p* < 0.001). Stress was positively correlated with heart rate (r(25) = 0.537, *p* = 0.006). Dental fear was correlated with anticipated pain (r(27) = 0.485, *p* = 0.010) and heart rate (r(25) = 0.569, *p* = 0.003). Correlations among psychological variables are illustrated in Figure 4.

### 3.2. Physiological Measures of Stress

The average heart rate of the participants was 84.4 bpm (SD = 12.6) with the highest heart rate measured at 112 bpm and the lowest being 64 bpm.

Saliva collection was completed on 31 (88.6%) participants, with 27 (87.1%) providing adequate quantity for analysis. Ultimately, salivary analysis was completed on 18 participants (13.3 ± 2.4 years old, 38.9% females). The remaining 9 samples were omitted from the final analysis due to invalid experimental results (i.e., inconclusive cortisol and alpha amylase measurements in the first randomly selected participants caused by dilution errors, as illustrated in Figure 2).

Salivary analyses of pediatric vs. orthodontic group related to normal reference ranges are presented in Table 4.

*Salivary cortisol.* Salivary cortisol (measured on n = 18 participants) had a mean of 0.02 μg/mL ± 0.02, with no statistically significant differences between pediatric and orthodontic patients (0.02 ± 0.02 vs. 0.03 ± 0.02, *p* = 0.731) nor between morning or afternoon collection (0.02 ± 0.02 vs. 0.03 ± 0.02, *p* = 0.994). Of 18 participants, 13 (72.2%) had a value outside the normal reference range of salivary cortisol, relative to the corresponding age group. Eight (44.4%, 62.5% from the pediatric clinic) of these had elevated levels of salivary cortisol. Of these 8 participants, 62.5% and 87.5% did not exhibit any dental anxiety or dental fear; however, 75.0% had moderate stress and 25.0% had high stress. Salivary cortisol levels did not significantly differ across sex and age groups (all *p*’s > 0.05).

*Salivary alpha amylase.* Salivary alpha amylase (measured on n = 16 participants) had a mean of 388.2 μg/mL ± 73.9, without exhibiting any statistically significant differences between pediatric and orthodontic patients (193.9 ± 109.0 vs. 64.6 ± 36.8, *p* = 0.072) nor between morning or afternoon collection (226.9 ± 109.0 vs. 149.6 ± 110.9, *p* = 0.304). All the obtained levels of alpha amylase were within normal ranges Alpha amylase levels were significantly higher among females compared to males (214.5 ± 121.7 vs. 101.7 ± 51.9, *p* = 0.040, Cohen’s *d* = 1.14).

### 3.3. Correlation Between Psychological and Acute Physiological Variables of Distress

None of the psychological variables were correlated with cortisol and alpha amylase (Figure 4). Although not significantly different, participants reporting greater stress symptoms had higher concentration of cortisol (0.02 ± 0.02 vs. 0.03 ± 0.00, *p* = 0.069). There was a statistically significant correlation between cortisol levels and stress severity in those with high cortisol (r(8) = 0.717, *p* = 0.045). None of the values of cortisol and alpha amylase significantly differed across participants with different severity of anxiety and fear (all *p*’s > 0.05). The incorporation of the three self-reported ad hoc questions measuring participants’ sleep quality, perceived stress, and dental fear showed no correlation amongst participants with physiological variables.

## 4. Discussion

For the current study, we employed both psychological questionnaires and salivary analyses to explore distress in pediatric patients undergoing dental procedures. While the validated self-reported questionnaires provide valuable insights into children’s perception of fear, anxiety, and stress [34,35], they remain subjective measures.

Studies report that about 30% of children have dental fear or anxiety when receiving dental treatment [19,36]. These numbers are consistent with our findings, where about 21.0% and 44.0% in our cohort expressed outcomes of dental fear and dental anxiety. Stress was rated even higher, reported by 85.3% (79.4% moderate stress and 5.9% high perceived stress) of the participants. These results highlight the utility and easy implementation of self-reported measures as a feasible screening tool for clinicians that can be repeated over time to monitor changes in psychological functioning in pediatric patients. Contrary to our expectation, no significant differences were observed in psychological distress between children undergoing dental procedures and those starting orthodontic treatment. Given the painless nature of orthodontic bonding—which does not require any local anesthesia nor the use of any vibrating instruments—we had anticipated lower distress scores. While 88.9% of orthodontic participants scored higher in moderate or severe stress combined compared to 84.0% of the pediatric patients expressing moderate stress, a lack of difference may also be attributed to the small sample size of the orthodontic group. The unequal group sizes reflected the 3:1 allocation ratio determined by clinic flow and organizational constraints. This imbalance reduced statistical power for between-group comparison. It may also be likely influenced by the fact that the PSS measures general stress rather than procedure-specific stress. Thus, these results should be interpreted with caution and considered preliminary.

Heart rate was positively correlated with anxiety and stress, consistent with other studies in the literature [37,38]. Given its simplicity and low cost, heart rate monitoring may represent a useful adjunctive measure in clinical practice to monitor levels of psychological distress [39], though further validation is required.

The analysis of salivary molecules provided additional insights but was constrained by the reduced number of usable samples (18 for cortisol, 16 for alpha amylase). Within this limited cohort, elevated cortisol values were observed in participants reporting higher stress symptoms, while alpha-amylase levels generally remained within reference ranges. Although alpha amylase appeared higher in females compared to males, no sex differences were detected in cortisol levels. Findings reported in the literature accounting for sex and age suggested that sex did not play any role in the concentration of cortisol levels in children under 14 years old [17,20]. However, another study by Alaki et al. claimed the opposite, showing that male children had higher salivary cortisol concentration than females [19]. This trend may be reflected in physiological data as well. For instance, a study by Ten Berge et al. found that female pediatric patients showed higher anxiety levels than males, based solely on physiological measures. [40]. This pattern appears to persist into adulthood, with several studies consistently reporting that adult females tend to experience greater anxiety than males [41].

Importantly, we did not find a significant correlation between psychological and physiological measures of distress. Few studies have investigated this correlation in the pediatric patients utilizing validated questionnaires [33,42], with a systematic review on this topic suggesting a weak correlation between objective and subjective measures [43]. For instance, salivary cortisol levels positively correlated with stress, fear and/or anxiety in few studies conducted in pediatric patients undergoing dental procedures [18,20,21,22]. Dental anxiety and cortisol were found to be significantly associated in studies that collected saliva samples across multiple timepoints [18,19]. Both studies found that cortisol levels peaked after the second appointment [18,19]. Thus, the fact that our study did not support a significant correlation could be attributed to the one-time point collection. Generally, the studies that supported a correlation between psychological and physiological measures of stress had larger sample sizes than the present study [18,20,21,22], while investigations with comparable sample sizes to our (between 20 and 30 participants) reported inconclusive results [17,44]. These discrepancies suggest that statistical power and methodological consistency play critical roles in detecting such associations.

Overall, our findings suggest that salivary biomarkers may reflect distinct aspects of the stress response-alpha amylase being more closely linked to sympathetic and cortisol to hypothalamic–pituitary–adrenal (HPA) axis activation [22]. However, given the limited sample size and methodological constraints, these results should be regarded as exploratory. Future studies with larger samples and standardized collection protocols are needed to clarify the relationship between psychological and physiological measures of distress in pediatric dental patients.

### Strengths and Limitations

This study has intrinsic limitations. First, salivary samples were collected at one timepoint prior to the dental procedures to limit the interruption of clinic flow, while prioritizing participants completion of the study. Although we originally planned to collect measures of physiological outcomes before and after the procedures, we limited our collection timepoint prior to start the dental appointment based on prior evidence showing higher cortisol levels before than after the dental appointment [20,45], which helped reduce timing-related variability and increased data completeness. Due to the university clinical workflows, we were unable to limit the time of day the appointments were scheduled. To minimize potential circadian bias, samples were collected both during morning and afternoon appointments, and no significant differences were found in salivary levels between these two groups. It is possible that the relatively small sample size and inter-individual variability limited our ability to detect circadian differences, or that procedure-related stress responses outweighed the expected diurnal variation. Future studies with larger cohorts and standardized sampling times are warranted to more definitely address this issue. Further, the final sample size of pediatric patients with usable salivary analysis was limited (n = 18) due to errors in dilution factors in the first eight samples. To mitigate selection bias, salivary analysis followed a randomized process, and a post hoc power analysis confirmed that the retained sample provided approximately 60% power to detect a larger effect size. Although invasive procedures such as pulpotomies or other dental procedures requiring local anesthesia and vibrating instruments are generally expected to elicit higher stress compared to non-invasive treatments [1,46], our analyses did not reveal differences in stress responses across the severity of the procedures performed. A potential reason could be that all study-related assessments were completed before treatment began. This suggests that, within the scope of our study, the invasiveness of the treatment was not a significant determinant of patient stress. Nonetheless, future research should standardize procedure type and use of anesthesia across participants, given the limited sample size of participants across procedure types in the present study. Finally, normalization of salivary molecules to total protein content may be influenced by local oral conditions, such as gingival inflammation or mucosal pathology, which were not systematically assessed in this study. Although we minimized confounding factors by excluding participants with oral infections and standardizing collection procedures, residual variability due to oral health status cannot be entirely excluded. Future studies incorporating measures of salivary flow rate, albumin, or clinical oral health indices may provide more refined approaches to normalization. While these limitations may affect the generalizability of the results, steps were taken at each stage to reduce bias and strengthen the validity of the findings.

Despite these limitations, this study has also several strengths. It is among the few in the pediatric literature that integrated and correlated psychological and physiological measures of distress. It analyzed a vast array of psychological and physiological salivary outcomes, providing valuable feasibility data for future larger-scale studies. It showed the clinical applicability of diverse salivary molecules in relation to various physiological outcomes.

## 5. Conclusions

In this cohort of pediatric patients undergoing dental procedures, 20.6% exhibited dental fear, 44.1% reported dental anxiety, and 85.3% presented moderate to high levels of stress. Salivary cortisol levels were above normal ranges in 44.4% of participants. No significant differences in psychological (dental fear, dental anxiety, and stress) or acute physiological measures of stress (salivary cortisol and alpha amylase) were observed between patients undergoing dental procedures and those starting orthodontic treatment, and no significant correlations were found between psychological and physiological outcomes. Although a proportion of participants with higher stress symptoms also showed elevated cortisol concentrations, these findings should be interpreted with caution given the limited sample size and lack of significant associations.

These preliminary data suggest potential avenues for future research, including studies with larger and more diverse pediatric populations, longitudinal designs to examine changes in psychological and physiological stress over time, and investigations into salivary biomarkers to better understand the relationship between psychological measures and physiological stress response.

## Figures and Tables

**Table 1:** Psychological outcomes and assessment tools.

Construct	Measure and Length	Answer Options	Scoring
Self-report stress symptomatology	13-item Perceived Stress Scale (PSS-C)	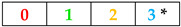 4-point Likert scale: never, a little, sometimes, a lot	Score range 0–39, being 0–13 = low stress, 14–26 = moderate stress, 27–39 = high perceived stress
Self-report dental fear symptomatology	15-item Children’s Fear Survey Schedule—Dental Subscale (CFSS-DS)	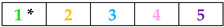 5-point Likert scale: From not afraid at all to very much afraid	Score range 15–75, with a score ≥38 consistent with clinical dental fear
Self-report dental anxiety symptomatology	8-item Modified Child Dental Anxiety Scale (MCDAS)	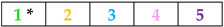 5-point Likert scale: from relaxed/not worried to very worried	Score range 8–40, being 8–19 = no anxiety, 19–30 = presence of anxiety, 31–40 = severe anxiety
Self-reported pain intensity	1-item Visual Analogue Scale (VAS)	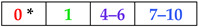 11-point scale: no pain, mild pain, moderate pain, severe pain	Score range 0–10, with 10 = worst imaginable pain
Self-reported sleep quality	1-item ad hoc question “How do you rate your quality of sleep last night?”	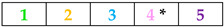 5-point Likert scale: worst quality to best quality	Score range 0–5, with 5 = best quality of sleep
Self-reported stress symptomatology	1-item ad hoc question “How do you rate your level of stress right now?”	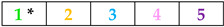 5-point Likert scale: no stress to extreme stress	Score range 0–5, with 5 = highest level of stress
Self-reported fear symptomatology	1-item ad hoc question “How do you rate your level of fear right now?”	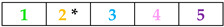 5-point Likert scale: not afraid to extremely afraid	Score range 0–5, with 5 = highest level of fear

* answer options that were most common amongst participants.

**Table 2:** Demographics of the included sample and comparison between pediatric and orthodontic patients.

Demographics	Total Sample N= 34	Pediatric Patients N = 25 (73.5%)	Orthodontic Patients N = 9 (26.5%)	*p* Value ^a^ (Effect Size)
Age (years, mean ± SD)	12.8 ± 2.7	13.4 ± 2.0	13.9 ± 1.4	1.00 (0.03)
Females (N, %)	18 (52.9%)	13 (52.0%)	5 (55.6%)	1.00 (0.03)
Race (N, %)				
Caucasian	27 (79.4%)	18 (72.0%)	9 (100.0%)	0.366 (0.31)
American Indian	1 (2.9%)	1 (4.0%)	0 (0.0%)	
African American	5 (14.7%)	5 (20.0%)	0 (0.0%)	
Prefer not to answer	1 (2.9%)	1 (4.0%)	0 (0.0%)	
Ethnicity (N, %)				
Hispanic	11 (32.4%)	6 (24.0%)	5 (55.6%)	0.111 (0.30)
Non-Hispanic	23 (67.6%)	9 (76.0%)	4 (44.4%)	

Note: SD = Standard Deviation. ^a^ results of chi-square tests (for categorical variables) and independent *t*-tests (for continuous variables) between pediatric and orthodontic patients.

**Table 3:** Psychological outcomes of the total sample and comparison between pediatric and orthodontic patients.

Variable	Total Sample N = 34	Pediatric Patients N = 25 (73.5%)	Orthodontic Patients N = 9 (26.5%)	*p* Value ^a^ (Effect Size)
**Psychological Variables**				
Dental Fear (CFSS-DS)				
Total Score (mean ± SD)	31.1 ± 9.6	32.7 ± 10.2	26.8 ± 6.0	0.128 (0.61)
No Fear (N, %)	27 (79.4%)	19 (76.0%)	8 (88.9%)	0.644 (0.14)
Clinical Dental Fear (N, %)	7 (20.6%)	6 (24.0%)	1 (11.1%)	
Perceived Stress (PSS-C)				
Total Score (mean ± SD)	23.8 ± 5.3	23.2 ± 6.0	25.6 ± 2.6	0.247 (0.46)
Low Stress (N, %)	5 (14.7%)	4 (16.0%)	1 (11.1%)	0.052 (0.42)
Moderate Stress (N, %)	27 (79.4%)	21 (84.0%)	6 (66.7%)	
High Perceived Stress (N, %)	2 (5.9%)	0 (0.0%)	2 (22.2%)	
Dental Anxiety (MCDAS)				
Total Score (mean ± SD)	18.9 ± 6.1	19.4 ± 6.8	17.3 ± 3.4	0.244 (0.34)
No Anxiety (N, %)	19 (55.9%)	13 (52.0%)	6 (66.7%)	0.591 (0.18)
Presence of Anxiety (N, %)	13 (38.2%)	10 (40.0%)	3 (33.3%)	
Severe Anxiety (N, %)	2 (5.9%)	2 (8.0%)	0 (0.0%)	
**Pain Variables**				
Pain before Procedure (mean ± SD)	0.5 ± 1.0	0.6 ± 1.1	0.0 ± 0.0	**0.015** (0.96)
Anticipated Pain (mean ± SD)	2.2 ± 2.5	2.1 ± 2.6	2.8 ± 2.1	0.505 (2.51)

Note: CFSS-DS: Children’s Fear Survey Schedule—Dental Subscale, MCDAS: Modified Children Dental Anxiety Scale, PSS-C: Perceived Stress Scale—Children. Significant differences are denoted with bolded font. ^a^ results of chi-square tests (for categorical variables) and independent *t*-tests (for continuous variables) between pediatric and orthodontic patients.

**Table 4:** Salivary analysis of pediatric vs. orthodontic group.

Salivary Molecule		Total Sample	Pediatric Patients	Orthodontic Patients	*p* Value ^a^
**Salivary Cortisol**	**(N, % of total sample)**	18 (52.9%)	13 (72.2%)	6 (33.3%)	
Mean Concentration (μg/mL)		0.02 ± 0.02	0.02 ± 0.02	0.03 ± 0.02	0.731
Below Reference (N, %)		5 (27.7%)	4 (22.2%)	1 (5.5%)	
Within Reference (N, %)		5 (27.7%)	3 (16.6%)	2 (11.1%)	
Above Reference (N, %)		8 (44.4%)	5 (62.5%)	3 (44.4%)	
**Salivary Alpha Amylase**	**(N, % of total sample)**	16 (47.1%)	12 (75.0%)	4 (25.0%)	
Mean Concentration (μg/mL)		338.2 ± 73.9	193.9 ± 109.0	64.6 ± 36.8	0.072
Below Reference (N, %)		0 (0.0%)	0 (0.0%)	0 (0.0%)	
Within Reference (N, %)		16 (100%)	12 (75.0%)	4 (25.0%)	
Above Reference (N, %)		0 (0.0%)	0 (0.0%)	0 (0.0%)	

^a^ results of independent *t*-tests between pediatric and orthodontic patients.

## Data Availability

The original contributions presented in the study are included in the article/Supplementary Materials; further inquiries can be directed to the corresponding author.

## Supplementary Materials

The following supporting information can be downloaded at https://www.mdpi.com/article/10.3390/children12091235/s1, A. Detailed Laboratory Protocols; B. Immunoglobulin A (IgA).
