# Supplementary material for: A Cross-Sectional Study of Salivary Cortisol, Alpha Amylase, and Measures of Psychological Distress in Children Undergoing Dental Procedures
Source: Children (Basel). 2025 Sep 16;12(9):1235. doi: 10.3390/children12091235 (PMC12468251; doi:10.3390/children12091235)
Supplement: Supplementary file 1 [file children-12-01235-s001.zip › children-3844097-supplementary.pdf]

Supplemental File A:

A. Detailed Laboratory Protocols

Bicinchoninic Acid (BCA) Assay

Analysis of BCA was completed to measure protein concentration amongst two Salivettes®. A widely adaptable method with manufacturer pilot protocol was used (ThermoFischer Scientific, Waltham, MA) and a 1:4 dilution with a 100 uL quantity was selected for salivary analysis. Protein standards consisted of diluted serum albumin (BSA) samples ranging from 0 ug/mL to 2,000 ug/mL. Working reagents (A+B) were a mixture of bicinchoninic acid and sodium carbonate in bottle A and copper (II) sulfate pentahydrate in bottle B. Standards, blanks (buffer) and saliva samples were plated onto 96-well plate in triplicates, to ensure reproducibility and validity of the findings. Subsequently, the samples were incubated for 30 minutes at 37°C to ensure accuracy of the plate reader. Absorbance was measured in Promega GloMax Discover plate reader (City, State) at 562nm. Standard curves were created by plotting the average standard versus its concentration in μg/mL. This allowed for each participant saliva samples to be converted into numerical values of total protein concentration which later was used to normalize results from the ELISA experiments.

Salivary Cortisol ELISA

Prior to assay, saliva samples were thawed on ice and kit contents were brought to room temperature (RT). Samples followed a 1:8 dilution factor and standards were prepped in reference to protocol which additionally included pre-coated 96-well plate (ThermoFischer Scientific, Waltham, MA). Cortisol standards were made via serial dilutions of the standard which contained organic solvent. Standards ranged from 0pg/ml to 3,200pg/ml. Standards, blanks (buffer) and saliva samples were plated into a pre-coated 96-well plate in triplicates followed by the addition of assay buffer (75 uL), cortisol conjugate (25 uL) and cortisol antibody (25 uL). Assay buffer has the ability to avoid detection of additional proteins. Cortisol conjugate and antibody participate in the competitive binding that takes place between the individuals' endogenous cortisol enzyme-linked cortisol during RT incubation for 60 minutes. RT incubation in salivary cortisol ELISA's is ideal for proper antibody-antigen binding. The primary result of these steps would have resulted in less conjugate binding if there was a significant endogenous cortisol quantity. A tetramethylbenzidine (TMB) substrate included within the kit was added to each well (100 uL) after incubation. An additional 30-minute incubation at 37°C after TMB serves to produce color changing results. The transition from clear to shades of blue is inversely correlated with the quantity of cortisol within the sample. Standards successfully completed showed darkest shade in correlation with lowest concentration. Notably, for cortisol, there is an inverse correlation with concentration and absorbance. Finally, 50 uL stop solution (1M Hydrochloric (HCl) Acid) was added, which addition changes the liquid mixture from blue to yellow, necessary for accurate readings. Readings on plate reader were completed within 10 minutes of stop solution being added to each well. The 96-well plate was placed in the Promega GloMax Discover plate reader and optical density was recorded at 450 nm.

Salivary Alpha Amylase ELISA

Alpha amylase assay followed similar protocol instructions as described above for cortisol. Saliva samples were thawed on ice and kit contents were brought to RT. As determined in pilot studies, samples required a dilution factor of 1:50,000 to quantify salivary alpha amylase due to its physiologically high concentration in saliva. Preparation of standards were based on protocol provided by the manufacturer (Biomatik, Ontario, Canada), which was made via serial dilutions of stock ranging from 0.156 pg/mL to 1,000 pg/mL.

Standards, blanks (buffer) and saliva samples were plated into a pre-coated 96-well plate in triplicates and incubated for 60 minutes at 37°C. Liquid was removed and the first detection reagent (A) was added to each well (100 uL) followed by a second incubation period of 60 minutes at 37°C. In addition to detection B (100 uL), a third incubation of 30 minutes at 37°C, TMB substrate solution (90 uL) was added to each well and a fourth incubation period (of 10-20 min, protected from light due to the TMB substrate solution) followed with wash steps in between. Same as cortisol, alpha amylase's final step consisted in the addition of 50 uL stop solution (1M HCl Acid) to each well. Readings were completed within 10 minutes of stop solution being added to each well. The 96-well plate was placed in the Promega GloMax Discover plate reader and optical density was recorded at 450 nm.

Normalization Process

The process of data normalization consisted of calculating the concentration of salivary molecules detected by the immunoassay experiments (cortisol, alpha amylase) as a function of the total protein concentration which was quantified through the BCA Assay. The purpose of including data normalization in our study was to properly correlate and make comparisons between participants. Dividing values extracted from saliva allowed us to numerically identify concentration values across saliva samples. Thus, expressing the salivary molecules concentration over total protein values indicates the total cortisol and alpha amylase values relative to each participant's total salivary protein content. Variability was taken into consideration amongst experiments on each protein between different saliva samples and corrected for any discrepancies. Most experiments required specific storing temperatures to prevent degradation and had estimated expirations. All concentrations were converted to ug/mL and were calculated to determine the exact value at fixed time (prior to dental procedure at T1) of cortisol and alpha amylase within participant's total salivary protein concentration.

B. Immunoglobulin A (IgA):

IgA was initially considered as a control biomarker due to its stability in children and role in immune function. However, preliminary experiments revealed inconsistent ELISA results, even after consulting the manufacturer and adjusting sample dilutions. Concentration values were often higher than BCA measurements of the same samples, making the data unreliable. Consequently, IgA was excluded from the final analysis and interpretation.

1. 2 Salivary Immunoglobulin A (IgA) ELISA:

Samples were diluted 1:5,000 and plated in triplicate on pre-coated plats (Novus Biologicals Centennial, CO). Stop solution was added to change the color from blue to yellow, and readings were taken within 10 minutes using a Promega GloMax Discover reader with chemiluminescence. Relative light unit (RLU) were used to calculate IgA concentrations via a standard curve.
